# A Study of Halide Ion Exchange-Induced Phase Transition in CsPbBr_3_ Perovskite Quantum Dots for Detecting Chlorinated Volatile Compounds

**DOI:** 10.1021/acsami.4c14868

**Published:** 2025-01-21

**Authors:** Chia-Chien Kuo, Duc-Binh Nguyen, Yi-Hsin Chien

**Affiliations:** † Department of Materials Science and Engineering, Feng Chia University, Taichung City, 40724, Taiwan

**Keywords:** phase transformation in perovskite, halide ions exchange, chlorinated gaseous detection, inorganic halide perovskite quantum dots

## Abstract

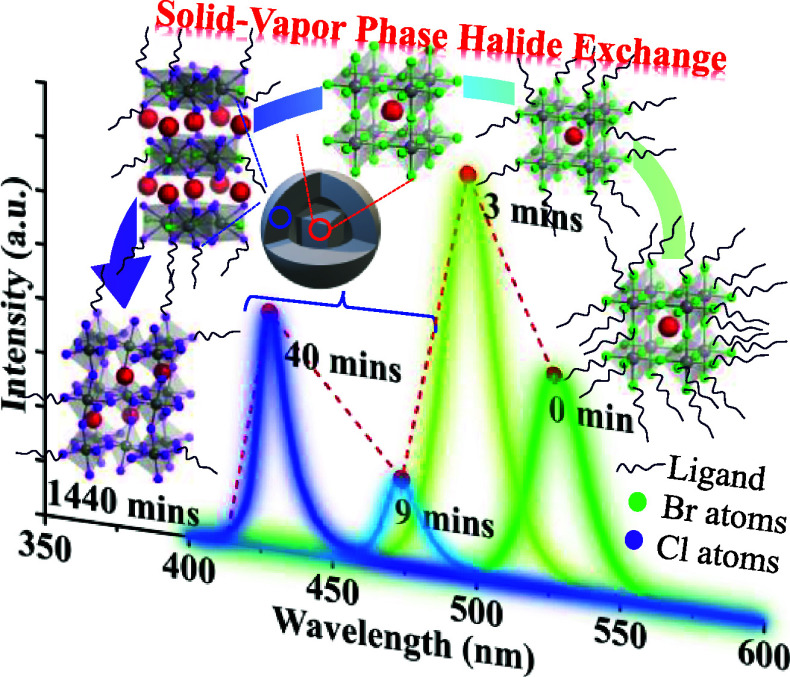

The unique optical properties of perovskite quantum dots (PQDs), particularly the tunable photoluminescence (PL) across the visible spectrum, make them a promising tool for chlorinated detection. However, the correlation between the fluorescence emission shift behavior and the interface of phase transformation in PQDs has not been thoroughly explored. In this study, we synthesized CsPbBr_3_ PQDs via the hot-injection method and demonstrated their ability to detect chlorinated volatile compounds such as HCl and NaOCl through a halide exchange process between the PQDs’ solid thin film and the chlorinated vapor phase. This exchange process, which occurs alongside chloride (Cl) and bromine (Br) ion exchange and halide atom rearrangement, leads to sequential structural changes: the initial CsPbBr_3_ cubic Pm3̅m phase transitions to the CsPb_2_Br_*x*_Cl_5–*x*_ tetragonal *I*4/*mcm* phase, which subsequently transforms into the CsPbBr_*x*_Cl_3–*x*_ orthorhombic *Pnma* phase. The detailed exploration of this proposed mechanism during chlorinated vapor detection with CsPbBr_3_ PQDs thin films, supported by X-ray diffraction (XRD) analysis and PL spectrum over time, revealed high sensitivity to HCl vapor. The limit of detection (LOD) for HCl vapor was determined to be 0.02 ppm in visual recognition and 0.005 ppm via PL spectra. Additionally, the LOD for NaOCl was established at 0.50 ppm, facilitated by the photolysis reaction accelerating the conversion of NaOCl to HCl vapor under UV light irradiation. These insights have enriched our understanding of the mechanisms involved and broadened the potential use of CsPbBr_3_ PQDs as PL detection probes for chloride ions.

## Introduction

Inorganic halide perovskite quantum dots (PQDs), denoted as CsPbX_3_ (where X = Cl, Br, I), exhibit several distinctive structural, optical, and electrical properties, including low trap densities, long carrier lifetimes, high photoluminescence (PL) quantum yields, and adjustable emission wavelengths.^1−4^ These properties position them as an attractive option for optoelectronic and sensor applications.^5−9^ The capability to alter crystal structure or composition under specified conditions, such as hydration/dehydration, gas adsorption/desorption, and ion intercalation, renders these substances relatively sensitive to external factors. Their remarkable sensitivity to temperature,^10−12^ humidity,^13,14^ and analytes, including toxic gases,^15−18^ metal ions,^19−21^ pesticides,^22−24^ and volatile organic compounds (VOCs),^25,26^ makes them possess significant potential for sensing applications. Even a minor modification in the crystal structure or morphology can result in alterations to the PL properties or charged potentials of halide PQDs, which can generate a response signal when detecting analytes.^27−29^ CsPbX_3_ is frequently employed as a PL detection probe due to its capacity to alter emission by adjusting the halide composition or partially substituting the cation site. Generally, the literature investigates the action of the internal energy and entropy in the exchange pathway of halide anions. They provide the hypothesis of anions exchanged processes to reveal the reduction of energy barrier on the formation of anion vacancies (Δ*E*_vacancy formation_). The internal anions tend to proceed spontaneously through vacancy diffusion, anion dynamic exchange, and construction of the PQDs.^30^ However, these processes are realized under the control of adjusting the PL emission and energy barrier, but the crystal structure and interface have not yet been investigated yet. In addition, PL detection operates based on the interaction between analytes and halide PQDs, which is expressed through three main types of signals: (i) emission peak shifting, (ii) PL quenching, and (iii) PL enhancement.^31−34^ These signals facilitate the rapid identification of analytes at a reduced cost compared with other conventional analytical techniques.

Chlorite ions play a pivotal role in both plant and animal life as well as in numerous human production activities. Exceeding the standards for chlorite content in domestic water, industrial wastewater, or the environment will have a detrimental impact on both the ecological balance and human health. Additionally, chlorite is a pervasive contaminant in human life, largely due to its colorless and odorless properties. While it plays a crucial role in disinfection processes, its potential health risks necessitate careful monitoring and regulation. Therefore, the rapid, precise, and reliable detection of the chlorite ion content is significantly important and has attracted considerable research interest. Conventional methods for detecting chlorite ions, such as electrochemical, chromatographic techniques, and inductively coupled plasma mass spectrometry, are typically expensive, necessitate complex instrumentation and procedures, and cannot be performed on site.^35−38^ Consequently, the inorganic perovskite quantum dots of CsPbBr_3_ PQDs are known for their high durability, simple manufacturing method, and high PL quantum yield, which has emerged as a promising alternative for overcoming these limitations. Moreover, CsPbBr_3_ PQDs probes for PL detection present a quick response and the ability to wavelength-shift easily to show obvious a color change when interacting with chlorite ions.^34,39−41^ They also have narrow emission line widths of 10–40 nm, which enhance sensitivity for chlorite detection. The halide anion exchange rate depends on the surface-limited, diffusion-limited, and displacement of halide atoms and vacancies within the structure. PQDs exhibit remarkable defect tolerance, with the dominating defect in the structure being halide vacancy, as their formation energy is lower than that of antisites and interstitial defects.^42,43^ This property facilitates the rapid migration of halide ions and vacancies within the PQD structure, thereby enabling efficient halide exchange even at low concentrations of chlorite ions. This phenomenon results in a notable enhancement of the response time and achieves a remarkably low detection limit. In addition, halide vacancies are considered active sites that enhance the sensitivity and selectivity of CsPbBr_3_ when used as a chlorinated vapor detection probe.^44,45^

Through these properties, some volatile chlorite ion solvents, including those found in daily life and industrial production, such as hydrochloric acid (HCl) and sodium hypochlorite (NaOCl), were selected to investigate the detection ability of the CsPbBr_3_ PQDs in this study. Thereafter, the hot-injection-prepared CsPbBr_3_ PQDs have been used to detect HCl and NaOCl via halide exchange. This study aimed to elucidate the phase transition process of CsPbBr_3_ PQDs through halide exchange with chlorite ions by combining X-ray diffraction (XRD) analysis, high-resolution transmission electron microscopy (HR-TEM), HR-TEM image simulation results, and fluorescence peak-shifting behaviors. We are the first to develop the construction of a comprehensive mechanistic model elucidating the correlation between the crystal structure rearrangement and PL emission. This model specifically addresses the anion exchange process occurring within the vapor phase of chloride and the solid phase of CsPbBr_3_ PQDs. Based on our proposed mechanism and chlorite ion detection strategy, the emission peaks of CsPbBr_3_ shifted from 520 nm (green color) to 430 nm (blue color) to 414 nm (purple color) during HCl vapor treatment, accompanied by the crystal structure transition from cubic (Pm3̅m phase) to orthorhombic (*Pnma* phase). Similarly, this mechanism for HCl vapor has facilitated the ongoing use of CsPbBr_3_ PQDs as PL detection probes for NaOCl, a chemical extensively used for disinfecting domestic wastewater and swimming pools. The monitoring NaOCl process is accelerated by a photolysis reaction under ultraviolet (UV) irradiation, inducing the chloride ions to exchange anions from CsPbBr_3_ to CsPbCl_3_. Among them, the CsPbBr_3_ PQDs probe exhibited a detection limit in HCl and NaClO of 0.005 and 0.50 ppm, respectively.

## Experimental Section

### Preparation of CsPbBr_3_ Perovskite Quantum Dots by the Hot Injection Method

CsPbBr_3_ PQDs were synthesized according to the method developed by Kovalenko and colleagues.^46^ Particularly, 1.00 g of cesium carbonate (Cs_2_CO_3_, 99% metals basis, Alfa Aesar), 2.5 mL of oleic acid (C_18_H_34_O_2_, 90%, Sigma-Aldrich), and 50 mL of 1-octadecene (C_18_H_36_, 90%, ACROS Organics) was loaded into a 100 mL three-necked flask and stirred at 120 °C for 40 min to remove all water in the solution. Subsequently, the solution was heated to 150 °C under a nitrogen environment until a clear solution was achieved, which typically took 60 min. The obtained cesium oleate was cooled and stored at room temperature until it was required for use. Note that it is necessary to heat the cesium oleate solution to 120 °C prior to use due to the low melting point of the solution, which would otherwise solidify at room temperature.^47,48^

0.69 g of lead bromide (PbBr_2_, 98%, Alfa Aesar), 5.5 mL of oleic acid (OA, 90%, Sigma-Aldrich), and 5.5 mL of oleyamine (OAm, 80–90%, ACROS Organics) were loaded to a 100 mL three-necked flask containing 50 mL of 1-octadecene and stirred at 120 °C for 40 min for water removal. After being heated to 180 °C for 10 min in a nitrogen environment, 3.5 mL of Cs-oleate solution was quickly added to the flask. 5 s later, the reaction was quenched by immersion in an ice bath. The PQDs were isolated via centrifugation at 10000 rpm for 10 min and directly diluted in toluene (C_6_H_5_CH_3_, 99.5%, J.T. Baker) for further characterization.

### Chloride Ion Exchange Reactions Involving PQDs and Chlorine-Containing Volatile Vapor

Detection of HCl vapor via a halide exchange process using CsPbBr_3_ PQDs. The concentration of the CsPbBr_3_ PQD solution was determined by inductively coupled plasma-optical emission spectrometry (ICP-OES). 400 and 100 ppm CsPbBr_3_ PQDs were drop-cast on a glass substrate with a size of 0.7 × 0.7 cm^2^ to form PQDs thin film. The CsPbBr_3_ PQD thin film was transferred onto the top of the vacuum testing vial.

To investigate the reaction mechanism, 1 mL of a HCl (37%, Honeywell Fluka) solution was added to the vacuum testing vial. The HCl was allowed to diffuse into the vial atmosphere and reach the PQD thin film. The PQD thin film was exposed for a period of between 3 and 1440 min before being removed for analysis. To detect HCl vapor, the HCl concentration was calculated and subsequently injected into a closed vessel containing a CsPbBr_3_ PQDs thin film, which was similar to the methodology employed by Zhu *et al.* with some modifications.^49^ The concentration of HCl vapor was controlled by evaporating 1 mL of a 37% HCl solution in a vacuum vial with a volume of 60 mL. The concentration of HCl vapor was determined using Aspen Plus (Version 12.1) software.^50^ A phase equilibrium calculation was performed to determine the concentration of HCl in the vapor phase. The electrolyte nonrandom-two-liquid (ELECNRTL) model was employed to estimate the mixture properties for the liquid phase, while the vapor phase was assumed to follow the Redlich–Kwong equation of state (RK-EOS). All of the built-in parameters in the Aspen database were utilized for the calculation. With these specifications, the HCl concentration was computed to be 293.852 ppm at a bubble point temperature of 300 K. Consequently, the bubble point pressure was established to be 0.207 bar. The various concentrations of HCl vapor were introduced into a 300 mL vacuum testing vial using an airtight syringe, which was then sealed. Following a 10 min reaction period, the PQD film underwent analysis.

### Detection of Sodium Hypochlorite via the Halide Exchange Process Using CsPbBr_3_ PQDs

The CsPbBr_3_ PQD (100 ppm) thin film was prepared in the same way as described above and transferred onto the top of a vacuum vial. A variety of sodium hypochlorite (NaOCl, Unilever) concentrations were introduced at the base of the chamber, where they were permitted to diffuse into the chamber atmosphere and reach the PQD thin film. It is worth noting that accelerating the decomposition of NaClO into chloride ions under UV light is an essential step in reducing the detection time of NaClO. After 40 min of reaction under UV light, the PQD thin film was taken and analyzed.

### Characterization

Absorption and photoluminescence (PL) spectra were acquired with a fluorescence spectrofluorometer (Edinburgh Instruments, model FS5). The concentration of PQDs was measured using an ICP-OES instrument (PerkinElmer OPTIMA 2000 DV). The PQDs’ morphology was examined using both a transmission electron microscope (TEM, JEOL JEM-1400(FLASH)) and a field emission transmission electron microscope (FE-TEM). A Bruker D8 Discover X-ray diffraction device with Cu Kα radiation (λ = 0.154 nm) was utilized to study the PQDs’ crystal structure. The chemical state of ions in PQDs was investigated using X-ray photoelectron spectroscopy (XPS, ULVAC PHI, Versa Probe 4). The XPS data were processed by using Elite software with a smart background baseline. Curve-fitting spin doublets split to ensure the area ratio of peaks corresponding to their orbitals. The quantification of the XPS peak was calculated based on the following eq (eq 1):^51^
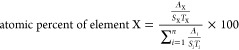
1where *A* is the area of the element, *S* is the sensitivity coefficient, *T* is the measurement time per data, and *n* is the number of regions. The sensitivity coefficients for Cs 3d, Pb 4f, Br 3d, and Cl 2p are 11.8, 9.0, 1.149, and 0.954, respectively.

## Results and Discussion

### Detection of HCl Vapor via the Halide Anion Exchange Process Using CsPbBr_3_ PQDs

**Figure 1:**
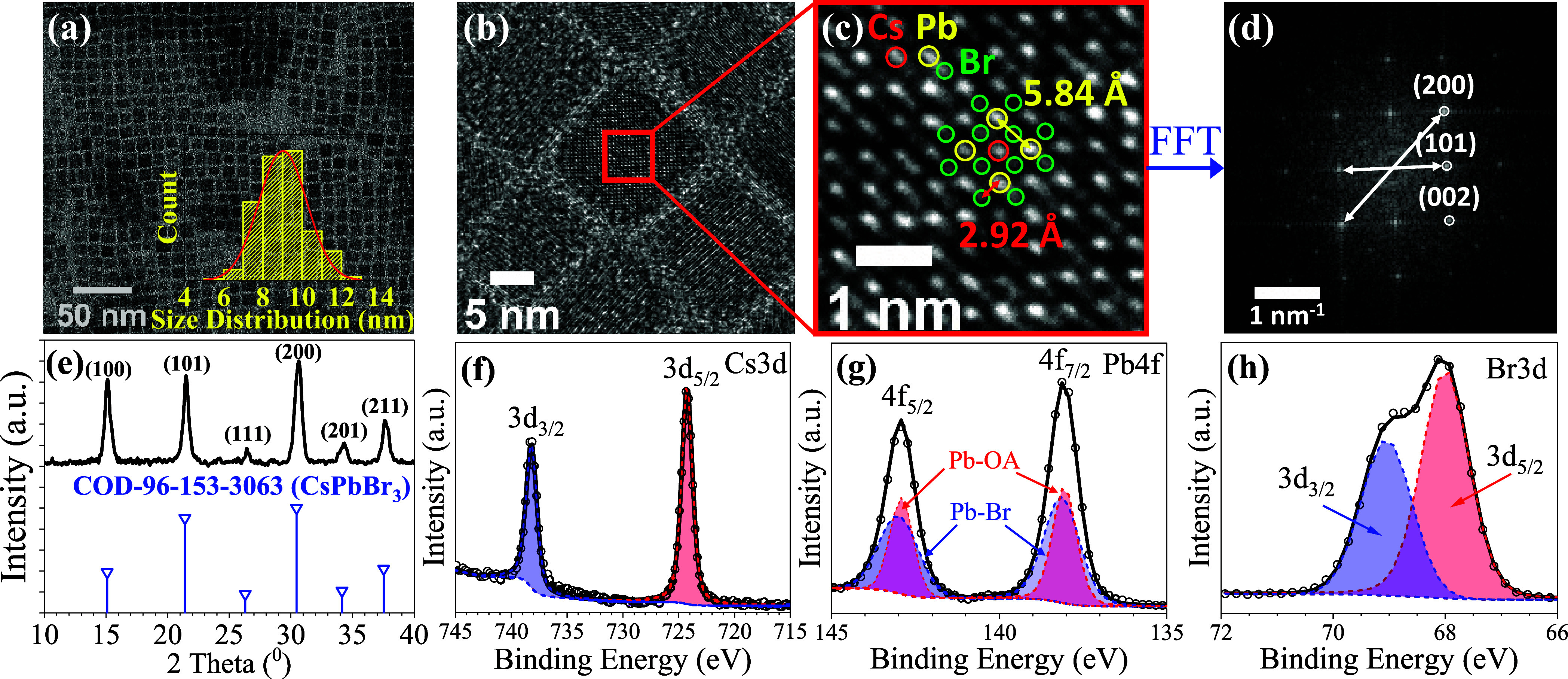
Characterization of CsPbBr_3_. (a) TEM image with size distribution insert. (b) HR-TEM image. (c) HR-TEM image of a single particle, represented by Cs, Pb, and Br atoms as red, yellow, and green circles, respectively. (d) The diffraction pattern and lattice fringe analysis. (e) XRD thin film pattern. The XPS analysis profile of (f) Cs 3d, (g) Pb 4f, and (h) Br 3d.

Cubic CsPbBr_3_ PQDs with a uniform size distribution of 9.02 ± 1.23 nm were successfully synthesized using the hot-injection method, as shown in Figure 1a. The high crystalline performance of the PQDs was confirmed through high-resolution transmission electron microscopy (HR-TEM) and fast Fourier transform (FFT) pattern analysis. The HR-TEM image of a single particle (Figure 1c) was obtained by focusing on cubic CsPbBr_3_ PQDs with the red square in Figure 1b, which revealed a remarkable crystalline structure with minimal atomic defects. Among them, the Cs and Pb atoms (represented by red and yellow circles, respectively) exhibited higher contrast than the Br atoms (depicted as green circles).^52^ The atomic positions of Cs, Pb, and Br aligned well with the unit cell structure of the CsPbBr_3_ cubic phase.^53^ The interatomic distances in the Pb–Pb and Pb–Br bonds were found to be 5.84 and 2.92 Å, respectively. Then, the bond angles of Br–Pb–Br and Pb–Cs–Pb were precisely determined at 90°, confirming the cubic perovskite structure of CsPbBr_3_ with crystallization in the Pm3̅m space group. In addition, the FFT pattern shown in Figure 1d was calculated from the image result of Figure 1c by using ImageJ software. The FFT pattern exhibits well-regulated arrayed spots of the cubic Pm3̅m phase, indicating the high purity of the cubic phase without twinning, dislocation, and stacking fault. The interplanar distances of the (101) and (200) planes were calculated to be 4.1 and 2.9 Å, respectively. Crystal orientation significantly affects the arrangement of the diffraction spots in the FFT pattern. Consequently, the FFT pattern of CsPbBr_3_ in the cubic Pm3̅m phase has been validated through simulation using ReciPro software, as illustrated in Figure S1.^54^ The FFT pattern aligned with the [010] direction index and its interplanar distance values of facets were estimated using Bragg’s law equation, showing consistency with our FFT analysis results (Figure S1b,d).^55^ The X-ray thin film diffraction (XRD) pattern in Figure 1e indicated that the peak intensity and position of 2θ = 15.13°, 21.50°, 26.46°, 30.61°, 34.27°, and 37.69° correspond to the (100), (101), (111), (200), (201), and (211) planes, respectively, which are well matched the crystallography open database (COD) number 96–153–3063 of the CsPbBr_3_ cubic Pm3̅m phase.^56^ The peak width indicated that the crystallite size was within the quantum confinement region. Moreover, the diffraction spots in the simulated FFT patterns (Figure S1d) completely coincide with the diffraction planes in the XRD pattern. The above findings demonstrate the consistent results presenting a pure phase of the CsPbBr_3_ cubic Pm3̅m phase. The XPS is employed to ascertain the chemical state of ions in CsPbBr_3_, as illustrated in Figure 1f–h. The formation of CsPbBr_3_ is demonstrated through the appearance of peaks corresponding to the Cs 3d, (Pb–Br)4f, (Pb-OA)4f, and Br 3d core-level at binding energies (BE) of 738.19 and 724.25 eV; 143.00 and 138.14 eV; 142.91 and 138.05 eV; and 69.05 and 68.00 eV, respectively.^57,58^ Additionally, the spin–orbit splitting values of Cs, Pb, and Br are 13.94, 4.86, and 1.05 eV, suggesting that the ions of Cs, Pb, and Br exist in the single valence state of +1, + 2, and −1, respectively.^59^

**Figure 2:**
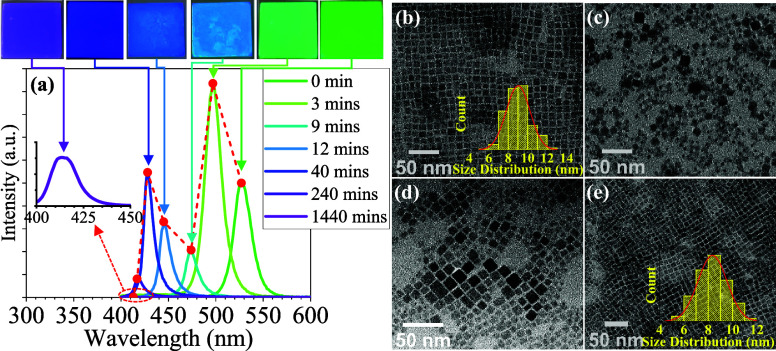
Halide ion exchange with various times of 0 to 1440 min through 293.852 ppm HCl_(g)_ with the CsPbBr_3_ PQD thin film (a) fluorescence spectrum and color change and emission peaks blue-shifting of under 365 nm UV light over time. The TEM images of (b) CsPbBr_3_ (0 min), (c) 9 min, (d) 40 min, and (e) 1440 min.

The ability to detect chloride-containing compounds was investigated by observing the emission peak shifts of CsPbBr_3_ PQDs during the halide exchange process between the HCl vapor phase and the solid state of CsPbBr_3_ PQDs. The optical stability properties of CsPbBr_3_ PQDs gain significant potential for obtaining a reliable sensing profile. The photoluminescence of the initial CsPbBr_3_ PQDs was investigated through absorption and PL spectra, as shown in Figure S2a. The CsPbBr_3_ PQDs exhibit an absorption peak at 496 nm, emission peak at 525 nm (λ_ex_ = 365 nm), and narrow-band emission featuring a bandwidth of 17.58 nm. Moreover, the CsPbBr_3_ PQDs presented stable fluorescence durability over 46 days, as illustrated in Figures S2b,c. Consequently, the HCl_(g)_ detection is demonstrated by the halide exchange between Br and Cl atoms, which are manifested through the color change and emission peaks blue-shifting of the CsPbBr_3_ PQDs thin film under 365 nm UV light over time, as depicted in Figure 2a. During the halide exchange process, the emission color of the PQDs thin film changed from green to sky-blue within 9 min, gradually turning to blue within 40 min and then to violet after 1440 min of the reaction under 365 nm hand-held UV irradiation. Accordingly, the fluorescence emission peak of PQDs thin film at 525, 474, 428, and 413 nm indicated a gradual replacement of Br atoms with Cl atoms in the PQD crystal lattice at 0, 9, 40, and 1400 min, respectively. After 1440 min, most of the Br atoms had been replaced with the Cl atoms, forming the pure CsPbCl_3_ PQDs, as evidenced by the emission peak at 413 nm.^60−62^ Then, we further discussed in detail the correlation between fluctuations in fluorescence intensity and morphological and crystal-phase changes via the subsequent XRD and TEM results during the halide exchange process. First, we investigate the morphological changes in PQDs over time throughout the halide exchange process by TEM images, as illustrated in Figure 2b–e. Among them, Figure 2b,c illustrates the evolution of the cubic structure in PQDs during the initial stage (0–9 min) of the halide exchange process. Figure 2c presents a slight structural decomposition instead of cubic morphology, illustrating the phenomenon involving the gradual Br ion substitution on the PQD surface with Cl ions, forming regions enriched with halide anions. This finding is attributed to the oleic acid ligands covered onto the PQD surface that play a role in maintaining the structure stability and are partially substituted by H^+^ ions from HCl vapor. This substitution results in the removal of the oleic acid from the structure and the insertion of the Cl ion into the surface Br vacancies (V_Br_) site, which leads to the PQD structural change, providing an increase in the emission intensity after 3 min (Figure 2a).^63,64^ Following 40 min, the halide exchange process gradually decelerates, facilitating the reconstruction of PQDs, which expels halide vacancies and reorganizes Cl ions within the structure, restoring it into a cubic form with an uneven size, as illustrated in Figure 2d. Finally, the PQDs ultimately revert to their original cubic form, exhibiting a uniform size distribution of 8.35 ± 1.20 nm after 1440 min, as shown in Figure 2e. The decrease in PQD size indicates that Cl ions have predominantly taken the place of Br ions, attributed to the smaller atomic radius of Cl ions compared to Br ions.

**Figure 3:**
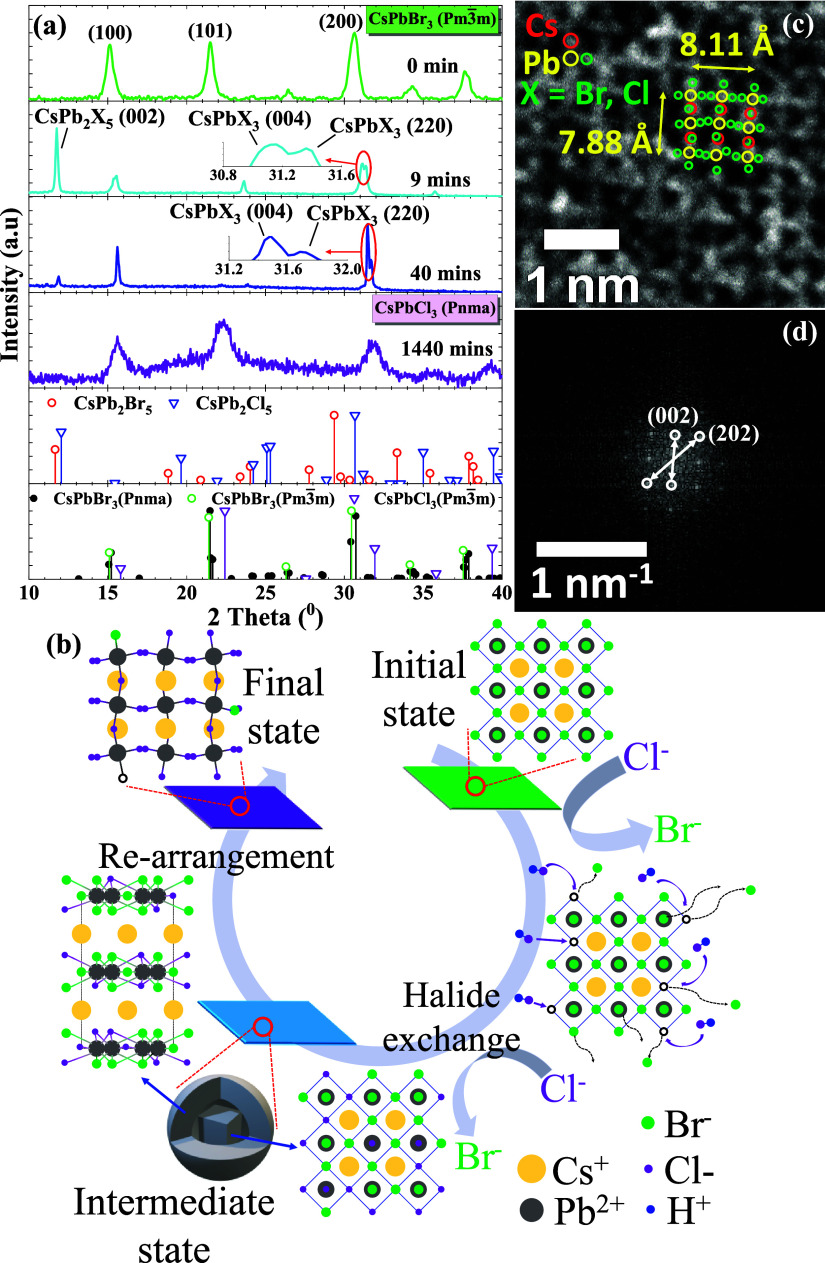
Crystal structures analysis over time of PQDs upon the halide exchange process through (a) the XRD pattern; (b) proposed mechanism of phase transformation; (c) HRTEM image of the final state after 1440 min; (d) fast Fourier transform (FFT) pattern obtained from HRTEM of (c) using the ImageJ software.

It is worth noting that the emission spectra are dependent on both the anion exchange kinetics and the phase transition rate. These processes occur simultaneously during the anion exchange process, resulting in complex alterations to the emission peak. However, the anion exchange kinetics and phase transition rate can be described with reasonable accuracy by combining the comparison of emission spectra and the XRD results. Simultaneously, the variety of crystal structures of PQDs has been carefully analyzed over time upon halide exchange through XRD analysis. As shown in Figure 3a, the cubic Pm3̅m phase of CsPbBr_3_ PQDs exhibit the diffraction peaks of 2θ at 15.13°, 21.50°, and 30.61°, representing crystal plane as (100), (101), and (200), respectively. The CsPbBr_3_ cubic phase transforms into a dual phase structure, including the CsPbBr_*x*_Cl_3–*x*_ and CsPb_2_Br_*x*_Cl_5-x_ phases, as evidenced by the appearance of peaks at 2θ ∼ 11.76°, 23.62°, and 35.74° in the XRD pattern after 9 min.^65−68^ The phase transition from the three-dimensional (3D) CsPbX_3_ structure to the two-dimensional (2D) CsPb_2_X_5_ structure due to the structure of the [PbX_6_]^4–^ octahedral (X represents Br and Cl) became more flexible and readily rotate. The combination of halide anion-rich regions with rotating [PbX_6_]^4–^ octahedral results in the initial phase transformed into the CsPb_2_X_5_ tetragonal *I*4/*mcm* phase (Figure 3a, at 9 min condition).^69^ Notably, a dramatic decrease in emission intensity of PQDs is affected by the phase transition from the cubic Pm3̅m phase to tetragonal *I*4/*mcm* phase upon 9 min of halide ion exchange (Figure 2a).^70,71^ Moreover, the XRD results after 9 min show the peak position at 2θ ∼ 15.48° and 31.12° is shifted closer to the CsPbCl_3_ standard spectrum position than the original CsPbBr_3_ PQDs sample, indicating that the exchange process between Br^–^ and Cl^–^ anions has occurred.^72,73^ The concentration gradient encourages Cl ions at the surface to diffuse into the core to form the CsPb_2_X_5_ phase. Upon 40 min, PQDs gradually reconstructed into a cubic form and transferred from the CsPb_2_X_5_ phase to the CsPbX_3_ phase, confirmed by the peaks decreasing intensity at 2θ ∼ 11.76°, 23.62°, and 35.74° on the XRD pattern. This XRD pattern characteristic of the CsPbX_3_ phase indicates that more Cl ions are present in the PQDs structure than Br atoms, illustrated by the peak right shift at 2θ ∼ 15.62° and 31.48°. Additionally, the peak at 2θ ∼ 31.48° has been deconvoluted into two subpeaks, thereby corroborating the existence of the CsPbX_3_*Pnma* phase.^74−76^ Simultaneously, these two subpeaks at peak position 2θ ∼ 31.48° and 31.71° on the XRD pattern become increasingly distinct, indicating the growth of the orthorhombic *Pnma* phase through 9 to 40 min.^71^ Although PQDs revert to the cubic morphology (Figure 2e), these peaks on the XRD pattern indicate the orthorhombic *Pnma* phase rather than the cubic Pm3̅m phase. After 1440 min, the peaks at 2θ ∼ 15.62°, 22.30°, and 31.84° have shifted toward the standard spectrum indicative of the CsPbCl_3_*Pnma* phase, which is supported by the XRD pattern. In addition, the full-width at half maximum peak (FWHM) of the XRD pattern is expanded to demonstrate the reduction in size and quantum confinement effect. Additionally, the rapid decrease in the emission intensity demonstrates the expeditious substitution of Cl ions into the structure after 1440 min (Figure 2a).

According to the above TEM results and XRD pattern, this is the first study to propose a detailed mechanism for the phase transition of CsPbBr_3_ PQDs followed by the interface reaction of halide exchange between the solid phase and gaseous hydrogen chloride, as shown in Figure 3b. During the halide exchange process, the CsPb_2_X_5_ tetragonal *I*4/*mcm* intermediate phase (where X represents Br and Cl) appears due to the flexible rotating [PbX_6_]^4–^ octahedral structure and excess halide atoms at the surface. The HCl vapor acts as an agent in reducing the energy barrier for phase transitions, similar to the role of water (H_2_O).^77,78^ The CsPb_2_X_5_ phase forms at the outer shell as the [PbX_6_]^4–^ octahedron becomes increasingly rigid and incompressible in halide anion-rich regions. This phenomenon induces the rotation and distortion of the [PbX_6_]^4–^ octahedral, leading to their transformation into [PbX_8_]^6–^. The occurrence of polyhedral distortions at the outer shell is necessary to release the internal lattice stress and maintain the rigid octahedral structure in the core. Among 2D structures, the AB_2_X_5_ configuration demonstrates the lowest formation energy compared to A_3_B_2_X_7_ and A_2_BX_4_.^79^ This suggests preferential formation of the CsPb_2_X_5_ phase over the other 2D structures. Surprisingly, the tetragonal *I*4/*mcm* phase undergoes continuous rotation and instantaneously transitions into the orthorhombic *Pnma* phase, resulting in reconstruction from [PbX_8_]^6–^ to [PbX_6_]^4–^ octahedra. In the final state, the formation of the orthorhombic *Pnma* phase was observed, rather than the cubic Pm3̅m phase. This behavior is attributed to the insufficient ligand outside, which prevented the reverting to the cubic Pm3̅m phase. Thus, the octahedral [PbX_6_]^4–^ reaches equilibrium within the CsPbX_3_ orthorhombic *Pnma* phase.^80,81^ Halide vacancies are identified as the dominant defects, requiring considerably lower formation energies for deeper traps such as antisites and interstitials.^43^ This dynamic encourages the migration of halide ions and vacancies more than other defects do. As a result, the CsPbX_3_ PQDs *Pnma* phase exhibits defect tolerance and facilitates the transport of halide vacancies to surface sites, thereby preserving structural stability from the interior to the exterior. Next, the HRTEM image (Figure 3c) indicates that the CsPbX_3_ in the final state has the form of the orthorhombic *Pnma* phase with lattice constants of *a* = *b* = 8.11 Å and *c* = 7.88 Å. The positioning of the spots around the Pb ions suggests that the [PbX_6_]^4–^ octahedral structure has been tilted and can not revert to its cubic Pm3̅m phase. Moreover, the FFT pattern obtained from Figure 3c using ImageJ software reveals the orderly spots characteristic of the orthorhombic *Pnma* phase, as depicted in Figure 3d. The interplanar distances of the (002) and (202) planes were calculated to be 3.9 and 2.8 Å, respectively. Furthermore, the HRTEM simulation image in Figure S3 demonstrated the orthorhombic *Pnma* phase with the lattice constant of *a* = *b* = 8.11 Å, a *c* constant of 7.88 Å, and a thickness of 8 nm. This further corroborates the hypothesis that the CsPbX_3_ compound exists as the orthorhombic *Pnma* phase in the final state.

The XPS results further discuss the alteration in binding energy (BE) and elemental composition of PQDs over time (0, 9, and 1440 min) in the halide exchange process under a HCl vapor environment, as illustrated in Figure S4 and Table 1. All XPS spectra were calibrated using the C 1s peak at 284.8 eV. The spin–orbital splitting and BE peaks of Cs, Pb, Br, and Cl in Table S1 remain constant over time at 13.94, 4.86, 1.05, and 1.60 eV, respectively, suggesting that ions of Cs, Pb, Br, and Cl exist in a single valence state of +1, + 2, −1, and −1, respectively.^82−84^ The initial composition of CsPbBr_3_ presents an X/Pb ratio of 2.70 and a Cs/Pb ratio of 0.56, suggesting that the atomic structural model of the CsPbBr_3_ surface is assumed to have a PbX_2_-terminated ([core][inner shell][outer shell] = [CsPbX_3_][PbX_2_][ligands]).^53^ The PbX_2_-terminated structure requires a significant amount of ligands to passivate lead surface atoms by impeding and disrupting Pb^2+^ octahedral coordination, characterized by the binding energy between Pb and oleate (Pb-OA).^58,85,86^ Therefore, the CsX-terminated is preferable to the PbX_2_-terminated structure.^87^ After the halide exchange process, the X/Pb ratios at 9 and 1440 min were 3.10 and 3.04, respectively, suggesting a transition in the atomistic structural model to the CsX-terminated due to the insufficiency of ligands to maintain the PbX_2_-terminated structure.^88^ Following a 9 min interval, the binding energies for the Cs 3d, (Pb – X) 4f, and Br 3d core levels shifted  to higher binding energies, with values of +0.1, + 0.52, and +0.39 eV, respectively, as shown in Figure S5. The shifts of the binding energy of Cs 3d and (Pb – X) 4f in the period 9 → 1440 min are significantly less than those in the period 0 → 9 min (Figure S5), indicating that the removal of ligands predominantly occurs during the initial stages of the reaction. The observed decrease in (Pb-OA) concentration of 3.02% after 9 min and 1.67% during the period 9 → 1440 min provides further corroboration of this conclusion. The  increased by 0.03 eV, indicating that Cl^–^ ions readily bond with Pb^2+^ to form (PbX_6_)^4–^ and (PbX_8_)^6–^. Additionally, the  decrease by 0.11 eV can be attributed to the relocation of Br ions to the surface, while Cl ions tend to move to the core. After 1440 min, the Pb metal existence was identified at BE levels of 136.30 and 141.16 eV, accounting for 1.09% of the total signal.^89−91^ This observation indicates that the sample decomposition results from halide vacancies at the surface, impeding the generation of Pb-halide binding in that area. The Cl ion concentration in the sample gradually increases over time, demonstrating the occurrence of a halide exchange process between the CsPbBr_3_ solid phase and the HCl vapor phase.

**Table 1:** XPS Results Including the Elemental Composition and Related Composition Percentage (%) of PQDs over Time (0, 9, and 1440 min) in the Halide Exchange Process under a HCl Vapor Environment

**elements**	**composition (%) of** 0 min	**composition (%) of** 9 min	**composition (%) of** 1440 min
**Cs**	13.14	15.57	18.36
**Pb-X**	13.41	13.57	14.58
**Pb-OA**	10.06	7.04	5.37
**Pb metal**	0.00	0.00	1.09
**Br**	63.39	36.13	1.61
**Cl**	0.00	27.69	58.99
**X/Pb**	2.70	3.10	3.04
**Cs/Pb**	0.56	0.76	0.92

**Figure 4:**
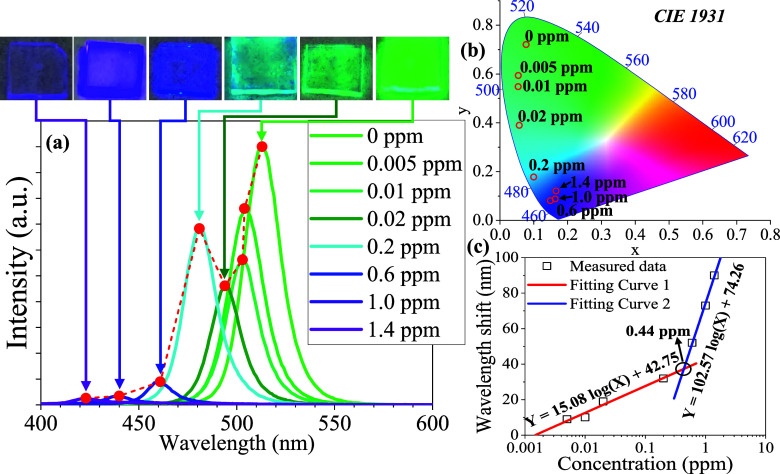
Evaluation of CsPbBr_3_ PQD thin film for various concentrations of HCl vapor detection through (a) fluorescence spectra and visual recognition; (b) CIE 1931 color space; (c) the relationship between wavelength shift and two HCl vapor concentration regions: 0.005–0.2 ppm and 0.6–1.4 ppm.

A detailed evaluation of halide ion exchange and phase transformation in CsPbBr_3_ PQDs is essential as the emission properties of PQDs are highly contingent upon their phase and stoichiometry, which is crucial to their detection capabilities. The investigation focused on assessing the capacity of the CsPbBr_3_ PQD thin film to detect HCl vapor by introducing varying concentrations of HCl vapor from 0 to 1.4 ppm. The results demonstrated that the CsPbBr_3_ PQD thin film exhibited sensitivity to HCl vapor and showed the potential for HCl vapor detection through photoluminescence (PL) and visual recognition, as depicted in Figure 4a,b. As followed by Figure S6, the CsPbBr_3_ PQD thin film was exposed to HCl vapor for 1, 10, and 15 min to ascertain the optimal detection time. The findings revealed that the 10 min induced obviously blue-shift wavelength comparison with 1 min upon 0.02 ppm HCl vapor. In Figure 4a, the visual recognition of the PQD thin film from lightly green-color to blue-green color was effective in detecting HCl vapor with a detection limit (LOD) of 0.02 ppm within 10 min. Then, the optimal visual operating range for CsPbBr_3_ PQDs thin films is determined to be between 0.02 and 1.0 ppm. These detection ranges could identify the tolerance value of HCl_(g)_ in the environment within 0.5 ppm to 5 ppm by regulations.^92^ However, the thin films appear nearly colorless, resulting in a limitation of visual recognition at 1.4 ppm. Therefore, the color of PQD thin films with various HCl vapor concentrations is clearly identified point by point by the CIE 1931 color coordinates (Commission Internationale de l’éclairage, CIE), as shown in Figure 4b. Accordingly, the emission peaks continuously blue shift with gradually increased HCl vapor concentration, whereas the LOD is 0.005 ppm from the emission spectrum. As shown in Figure 4a, the fluorescence emission intensity decreased due to the slow phase transition rate of CsPbBr_3_ PQDs. This is attributed to the phase transition from the cubic Pm3̅m phase to the tetragonal *I*4/*mcm* phase with the 2D CsPb_2_X_5_ state at low concentrations of HCl vapor (0.005–0.02 ppm) after 10 min reaction. When exposed to HCl vapor concentrations ranging from 0.02 to 0.2 ppm, the fluorescence intensity of the CsPbBr_3_ PQDs thin film dramatically increased, and its color shifted to sky-blue. This change is attributed to the increased diffusion of Cl ions into the crystal structure, which leads to a phase reconstruction. As the concentration of HCl vapor increases, the reaction rate escalates, causing a rapid blue-shift of the emission peaks toward the violet region. At 1.4 ppm HCl vapor, the PQD thin film appears to lower emission intensity due to the low photoluminescence quantum yield (PLQY) of CsPbCl_3_ and the presence of numerous defects in the final state. These defects are attributed to the high concentration of H^+^ ions, which attack the ligand sites of oleic acid. The above-observed alteration in the trend of emission peaks is entirely consistent with the emission properties depicted in Figure 2a and the proposed mechanism illustrated in Figure 3b. Moreover, the relationship between the wavelength shift and concentration had a strong linear correlation according to two different concentration regions, as depicted in Figure 4c. The reaction rate at HCl concentrations below 0.44 ppm is slower compared to concentrations above 0.44 ppm. For concentrations below 0.44 ppm, the linear regression equation is *Y* = 15.08 log(*X*) + 42.75, with a correlation coefficient of *R*^2^ = 0.9673. According to the above linear equation, the LOD of the HCl vapor can be determined to be 0.0014 ppm. For concentrations ranging from 0.44 to 1.4 ppm, the linear regression equation is *Y* = 102.57 log(*X*) + 74.26, with a correlation coefficient of *R*^2^ = 0.9967.

### Detection of Sodium Hypochlorite via the Halide Ion Exchange Process Using CsPbBr_3_ PQDs

Based on the aforementioned halide ion exchange process between CsPbBr_3_ PQDs and Cl ions, we evaluated another related chlorinated toxic vapor, sodium hypochlorite (NaOCl), which is a strong oxidizing agent capable of causing damage to the skin or respiratory tract upon contact. The development of an on-site NaOCl sensor is essential because NaOCl is commonly utilized for household bleaching and disinfection of municipal water and wastewater, swimming pools, and the food industry. For the NaOCl aquation detection, the 925 ppm NaOCl solution was introduced into a vacuum chamber preplaced with a CsPbBr_3_ PQDs thin film. At ambient temperatures, NaOCl can simultaneously form HOCl_(g)_ according to the following chemical reaction equations (eqs 2 and 3):^93^
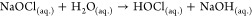
2
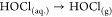
3

Thereafter, HOCl_(g)_ reacts with both oleic acid and oleylamine ligands onto PQDs to form 9-chloro-10-hydroxyoctadenoic acid, 10-chloro-9-hydroxyoctadecanoic acid, monochloramine, and dichloramine.^94^ Then, monochloramine and dichloramine decompose to form HCl vapor, which is detectable by CsPbBr_3_ PQDs via a vapor–solid phase halide ions exchange reaction.^95,96^ As shown in Figure S7, the results indicate a 32 nm blue shift in the emission peak during the 16 h exchange reaction. This is due to several intermediate reactions, which cause the halide ion exchange reaction rate to be slow in the NaOCl aqueous detection. Therefore, UV light (λ = 365 nm) was introduced as an accelerator to speed up the detection, resulting in the HOCl vapor being directly transformed into H^+^ and Cl^–^ ions through the following reaction equation (eq 4):^97−99^
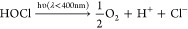
4

**Figure 5:**
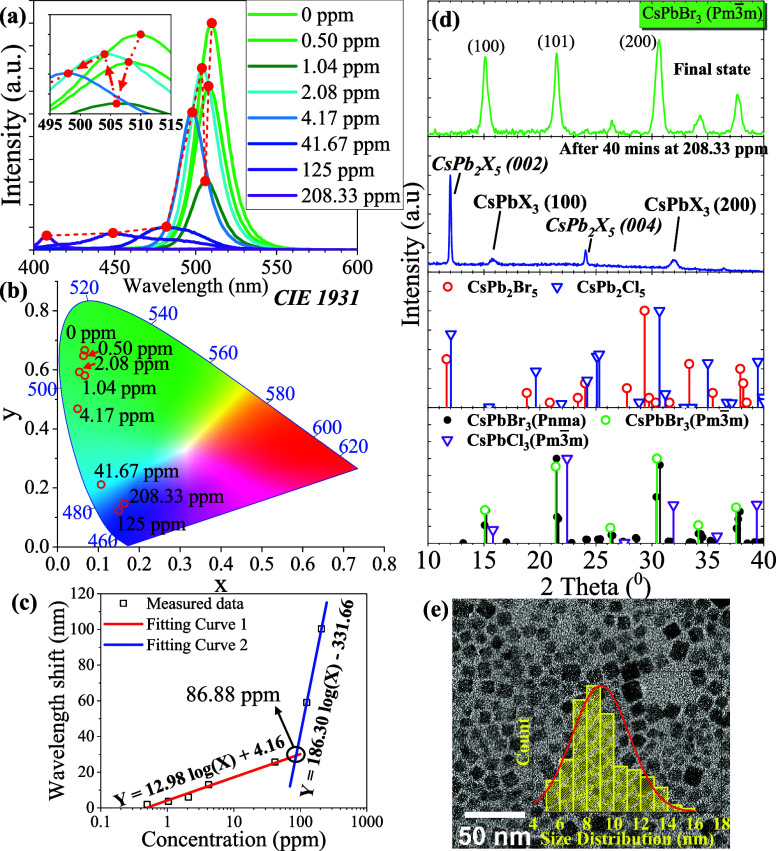
Evaluation of CsPbBr_3_ PQD thin film for NaOCl detection at 40 min reaction under UV light irradiation through (a) various concentrations of NaOCl detection from 0 to 208.33 ppm determined by fluorescence spectrum; (b) CIE 1931 color space; (c) the relationship between wavelength shift and two NaOCl_(aq)_ concentration regions: 0.5–41.67 ppm and 125–208.33 ppm; (d) the XRD pattern analysis profile of CsPbBr_3_ PQDs and at 208.33 ppm of NaOCl; (e) TEM image of final state.

Based on eq 4, the halide ion exchange reaction can be significantly accelerated by UV irradiation, resulting in emission peaks that are easily distinguishable by PL spectra and CIE color space at different concentrations of NaOCl after 40 min, as shown in Figure 5a,b. The results indicate that CsPbBr_3_ PQD thin films are highly sensitive to detecting NaOCl. The observed modifications in emission intensity suggest that the halide exchange mechanism between the CsPbBr_3_ PQD thin film and NaOCl is analogous to the mechanism occurring during halide exchange with the HCl vapor. In the inset spectrum of Figure 5a, the blue shift in wavelength of the emission peak is relatively insignificant, and then the fluorescence intensity exhibits a dramatic decrease when the concentration of NaOCl falls below 2.08 ppm, indicating a comparatively slow exchange rate between Br and Cl ions. Moreover, the emission intensity increased above 2.08 ppm owing to the greater amount of Cl ions diffusion into the crystal structure and a phase reconstruction. The LOD can be determined to 0.5 ppm by the emission spectrum upon UV light irradiation. The linear regression of the wavelength shift versus concentration is shown in Figure 5c. The linear regression equation for concentrations below 86.88 ppm is *Y* = 12.98 log(*X*) + 4.16 with a correlation coefficient of *R*^2^ = 0.9742, while the linear regression equation for concentrations from 86.88 to 208.33 ppm is *Y* = 186.30 log(*X*) – 331.66. The correlation between wavelength shift and analyte concentration indicates that the halide exchange rate is faster with HCl than with NaOCl. Notably, the XRD pattern exhibits an intermediated dual-phase of CsPbBr_*x*_Cl_3–*x*_ cubic Pm3̅m phase and CsPb_2_Br_*x*_Cl_5-x_ tetragonal *I*4/*mcm* phase after 40 min of reaction at the highest concentration of 208.33 ppm, indicating that this halide ion exchange reaction did not reach completion to the final state, as illustrated in Figure 5d. Furthermore, the particle size of the PQDs exposed to 208.33 ppm NaOCl for 40 min measured 9.05 ± 2.14 nm, which is approximately the same as the original CsPBr_3_ PQDs. This result suggests that the Cl ions have not completely substituted the Br ions and that the reaction has only stopped at the intermediate phase.

**Figure 6:**
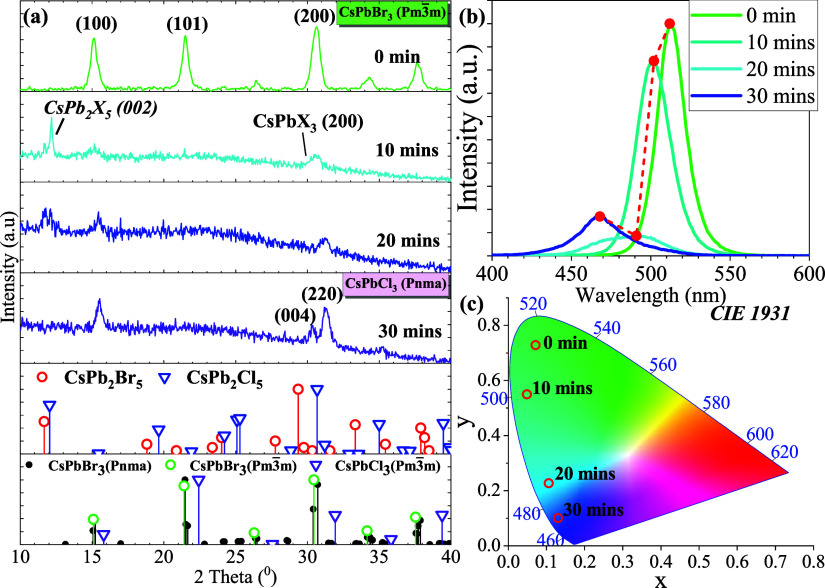
Evaluation over time of CsPbBr_3_ PQDs thin film and NaOCl 5% detection at 10, 20, and 30 min under UV light irradiation through (a) XRD analysis profile; (b) fluorescence spectrum; (c) CIE 1931 color space.

To investigate if the halide ion exchange process between the CsPbBr_3_ PQDs thin film and NaOCl under UV light follows the after-mentioned mechanism while the halide exchange under HCl vapor conditions, the CsPbBr_3_ PQDs thin film was subjected to a vial containing 5% NaOCl for further analysis via XRD and PL at various intervals. As shown in Figure 6a, the XRD pattern reveals that CsPbBr_3_ undergoes a phase transformation overtime at 10, 20, and 30 min. After 10 min of reaction, the initial CsPbBr_3_ cubic Pm3̅m phase transforms into a dual phase consisting of CsPbX_3_ cubic Pm3̅m phase and CsPb_2_X_5_ tetragonal *I*4/*mcm* phase. The evidence of transformation is substantiated by the emergence of a peak at 2θ ∼ 12.17° of the XRD pattern for the (002) plane, indicative of the presence of the CsPb_2_X_5_ tetragonal *I*4/*mcm* phase. Additionally, both peak intensities reduce at 2θ ∼ 15.13° and 30.61°, presenting the (100) and (200) lattice planes of the CsPbX_3_ cubic Pm3̅m phase. Notably, the peak positions of (100) and (200) planes remain almost unchanged in comparison to the initial CsPbBr_3_ cubic Pm3̅m phase spectral, indicating a slow rotation speed of the octahedra in the phase transformation upon NaOCl detection. Upon 20 min, a noticeable intensity decrease in the dominant peak at 2θ ∼ 12.17° for CsPb_2_X_5_ was observed, indicating the gradual transformation of the CsPb_2_X_5_ tetragonal *I*4/*mcm* phase to the CsPbX_3_ orthorhombic *pnma* phase. In addition, the peak at 2θ ∼ 31.5° on the XRD pattern was determined to be the CsPbBr_*x*_Cl_3–*x*_ orthorhombic *pnma* phase, illustrating a higher percentage of Cl ions in the PQDs structure. Finally, the entire transition from the CsPb_2_X_5_ tetragonal *I*4/*mcm* phase to the CsPbX_3_ orthorhombic *pnma* phase was confirmed by the disappearance of the (002) plane of the CsPb_2_X_5_ tetragonal *I*4/*mcm* phase. This was accompanied by the splitting of the (002) plane of the CsPbBr_3_ cubic *Pm*3̅*m* phase into two distinct peaks at approximately 30.37° and 31.26°, corresponding to the (004) and (220) planes of the CsPbX_3_ orthorhombic *pnma* phase. Moreover, the observed wavelength shift in the photoluminescence results suggests that the halide ion exchange process in NaOCl occurs at a slower pace compared to that in HCl, as illustrated in Figures 6b,c. The initial decrease in emission intensity during the first 20 min followed by an increase after 30 min, indicates that the 2D CsPb_2_X_5_ tetragonal *I*4/*mcm* phase forms initially and then fully transitions to the CsPbX_3_ orthorhombic *pnma* phase. Notably, these observations are consistent with the proposed mechanism for the halide exchange process between the solid phase of the CsPbBr_3_ PQDs and the gaseous Cl ions.

## Conclusions

This study highlights the potential of CsPbBr_3_ PQDs for detecting chlorinated volatile compounds such as HCl and NaOCl, through a halide exchange process. Significantly, the CsPbBr_3_ PQD thin films demonstrated high sensitivity to HCl with an LOD of 0.02 ppm for visual recognition and 0.005 ppm in PL spectra, while the LOD for NaOCl was 0.50 ppm under UV light irradiation. Notably, this exchange process induces structural phase transitions from the 3D CsPbBr_3_ cubic Pm3̅m phase to the 2D CsPb_2_Br_*x*_Cl_5–*x*_ tetragonal *I*4/*mcm* phase and finally to the CsPbCl_*x*_Br_3–*x*_ orthorhombic *Pnma* phase. The results of fluctuation emission intensity and TEM image indicated the occurrence of the intermediate tetragonal *I*4/*mcm* phase and structural rearrangement phenomenon to the orthorhombic *Pnma* phase during the halide exchange process. Furthermore, the XRD analysis profile, HR-TEM image, and fast Fourier transform (FFT) analysis also supported the final orthorhombic *Pnma* phase, revealing that halide vacancies are the dominant defects in maintaining structural stability from the interior to the exterior. These findings enhance the understanding of the mechanisms involved in the halide exchange process in PQDs and broaden the potential use of CsPbBr_3_ PQDs as PL detection probes for chloride ions, highlighting their application in environmental monitoring and other fields requiring the sensitive detection of chlorinated gases.

## Supporting Information Available

3D structure of CsPbBr_3_ cubic Pm3̅m phase; a view direction of the CsPbBr_3_ cubic Pm3̅m phase for the recalculated HR-TEM image, and the FFT pattern; the optical properties of the CsPbBr_3_ cubic Pm3̅m phase and their PL stability; the recalculated HR-TEM image and FFT pattern of the CsPbCl_3_ orthorhombic *Pnma* phase; the XPS results and the shifts of the binding energy for various core levels by the time of the CsPbBr_3_ PQDs thin film; the PL results for the CsPbBr_3_ PQDs thin film after being exposed to HCl gas and NaOCl (PDF)
