# Supplementary material for: A Study of Halide Ion Exchange-Induced Phase Transition in CsPbBr3 Perovskite Quantum Dots for Detecting Chlorinated Volatile Compounds
Source: ACS Appl Mater Interfaces. 2025 Jan 21;17(4):7043–55. doi: 10.1021/acsami.4c14868 (PMC11788985; doi:10.1021/acsami.4c14868)
Supplement: Supplementary file 1 — am4c14868_si_001.pdf [file am4c14868_si_001.pdf]

Supporting Information

A study of Halide Ion Exchange Induced Phase Transition in $CsPbBr_3$ PQDs for Detecting Chlorinated Volatile Compounds

*Chia-Chien Kuo[a], Duc-Binh Nguyen[a], Yi-Hsin Chien[a,]**

[a] Department of Materials Science and Engineering, Feng Chia University, Taichung City, 407001, Taiwan

* Email: yhchien@fcu.edu.tw

CONTENTS

Figure S1 – (a) 3D structure of $CsPbBr_3$ cubic $Pm\bar{3}m$ phase. (b) A view direction of the $CsPbBr_3$ cubic $Pm\bar{3}m$ phase for (c) The HR-TEM image, and (d) the FFT pattern, which were recalculated based on the $CsPbBr_3$ cubic $Pm\bar{3}m$ phase with the lattice constant of 5.84Å, an average size of 9 nm and the crystal orientation as shown in Figure 1b using ReciPro.

Figure S2 – (a) The optical properties of $CsPbBr_3$ cubic $Pm\bar{3}m$ phase and (b, c) their PL stability.

Figure S3 – (a) The HR-TEM image, and (b) FFT pattern were recalculated based on the $CsPbCl_3$ orthorhombic Pnma phase with the lattice constant of a = b = 8.11 Å and c = 7.88 Å, an average size of 8 nm and the crystal orientation as shown in Figure 3c using ReciPro.

Figure S4 – The XPS results of the $CsPbBr_3$ PQDs thin film exposed to HCl gas at varying intervals.

Figure S5 – The shifts of the binding energy for various core levels by the time.

Figure S6 – The PL results for the $CsPbBr_3$ PQDs thin film, which was exposed to 0.02 ppm of HCl gas for 1, 10, and 15 minutes to identify the optimal detection time.

Figure S7 – The PL results for the $CsPbBr_3$ PQDs thin film, which was exposed to 5% of NaOCl without UV-acceleration for 16 hours,

Table S1 – Binding energy, full width at half maximum (FWHM), and spin-orbital splitting of Cs3d, Pb4f, Br3d, and Cl2p of $CsPbBr_3$ after exposed with HCl gas for 0, 9, and 1440 minutes.

Table S2 – Recently reported nanomaterial-based methods for detection of HCl gas.

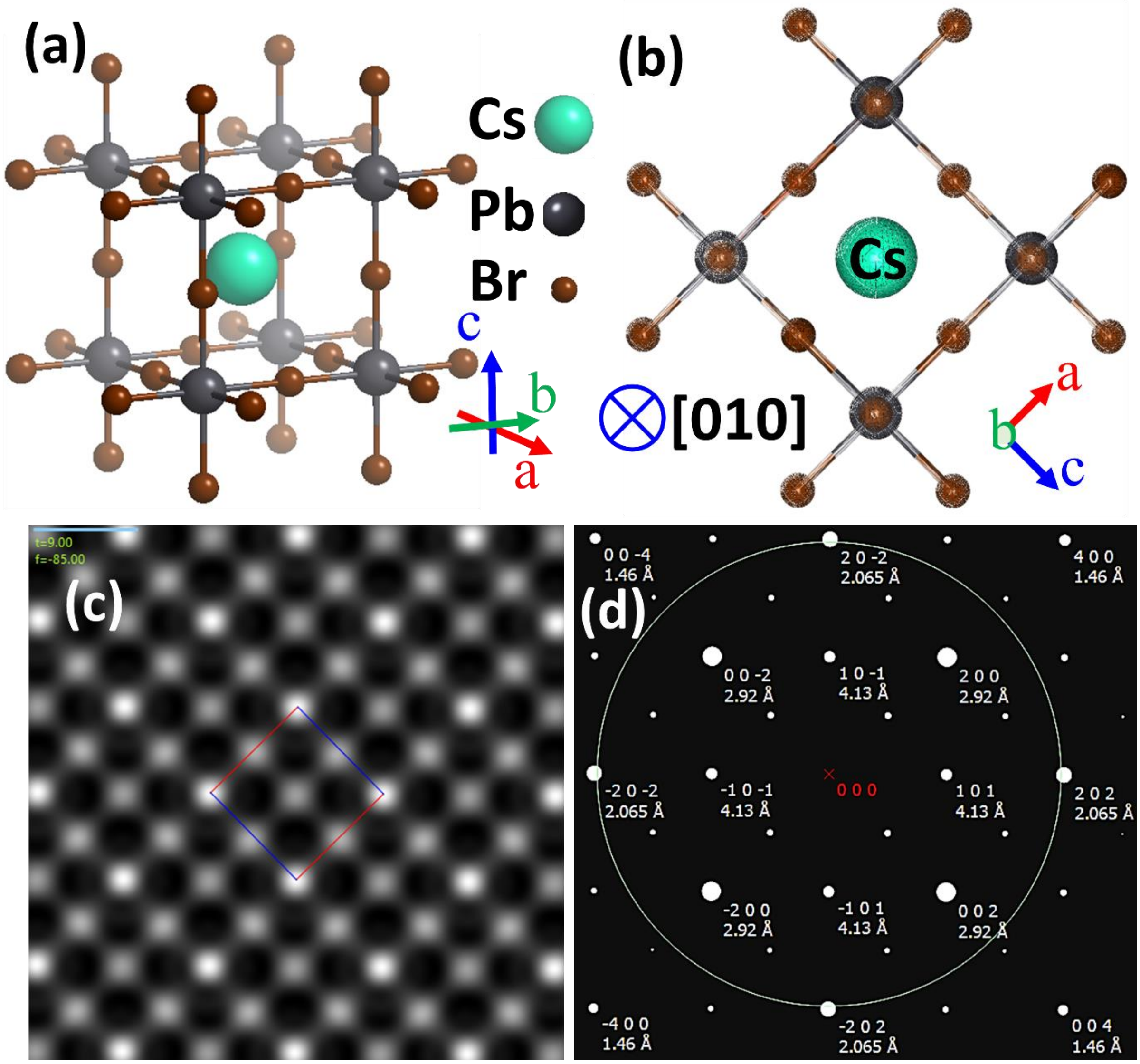

Figure S1. (a) 3D structure of $CsPbBr_3$ cubic Pm$\overline{3}$m phase. (b) A view direction of the $CsPbBr_3$ cubic Pm$\overline{3}$m phase for (c) The HR-TEM image, and (d) the FFT pattern, which were recalculated based on the $CsPbBr_3$ cubic Pm$\overline{3}$m phase with the lattice constant of 5.84Å, an average size of 9 nm and the crystal orientation as shown in Figure 1b using ReciPro.

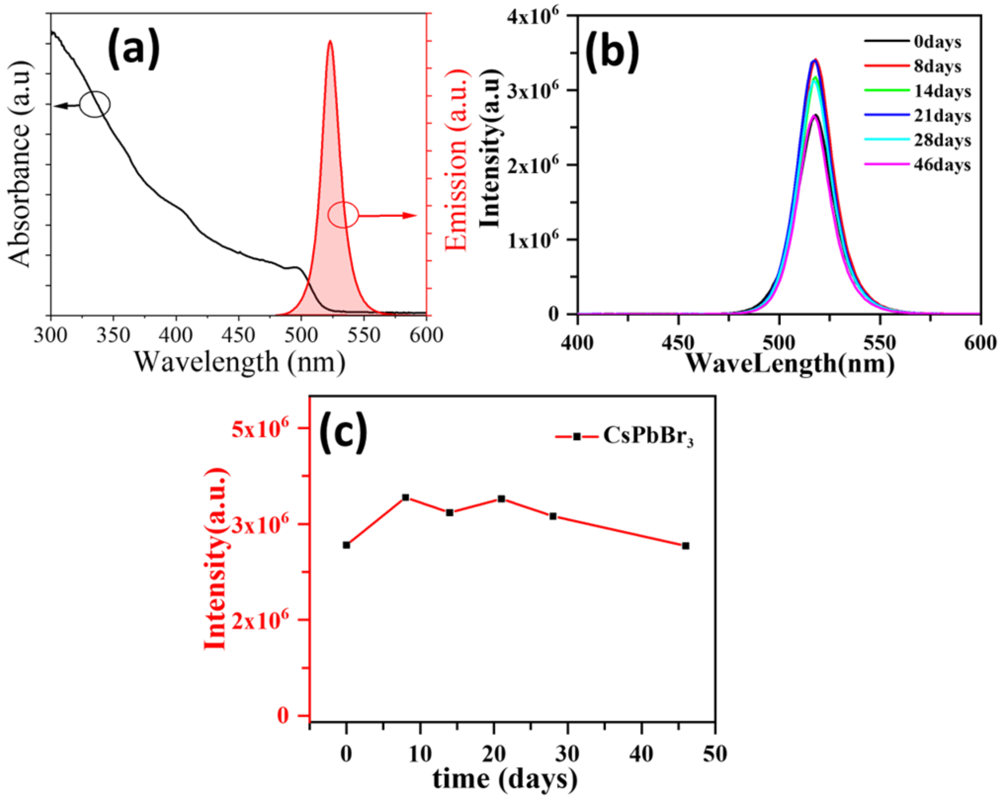

Figure S2. (a) The optical properties of $CsPbBr_3$ cubic $Pm\bar{3}m$ phase and (b, c) their PL stability.

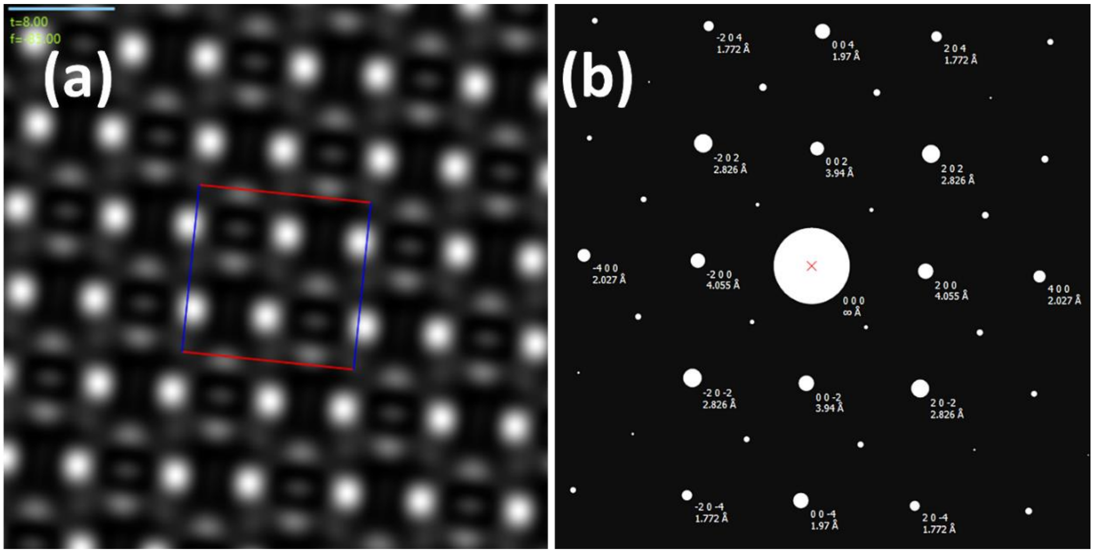

Figure S3. (a) The HR-TEM image, and (b) FFT pattern were recalculated based on the $CsPbCl_3$ orthorhombic Pnma phase with the lattice constant of a = b = 8.11 Å and c = 7.88 Å, an average size of 8 nm and the crystal orientation as shown in Figure 3c using ReciPro.

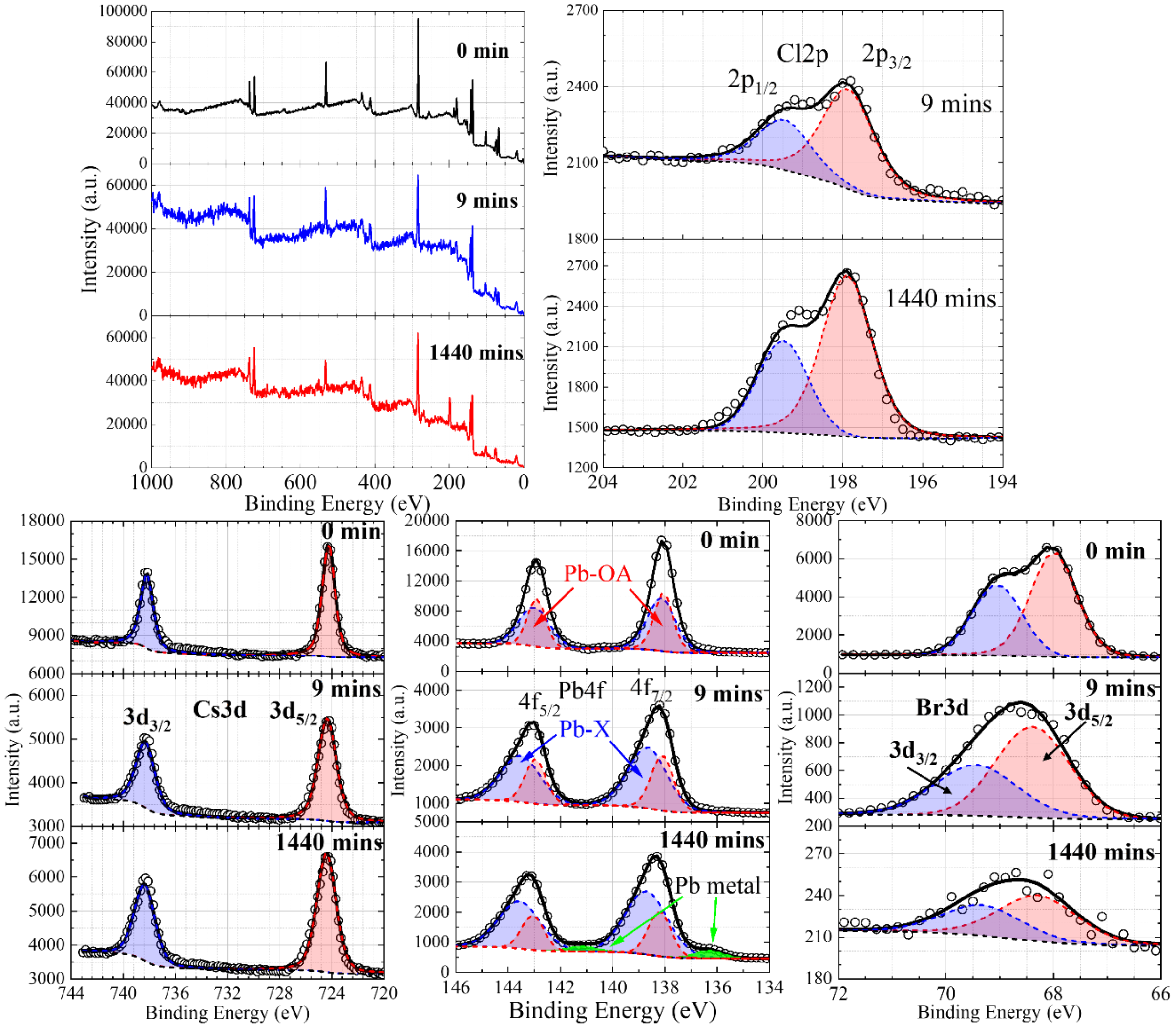

Figure S4. The XPS results of the $CsPbBr_3$ PQDs thin film exposed to HCl gas at varying intervals.

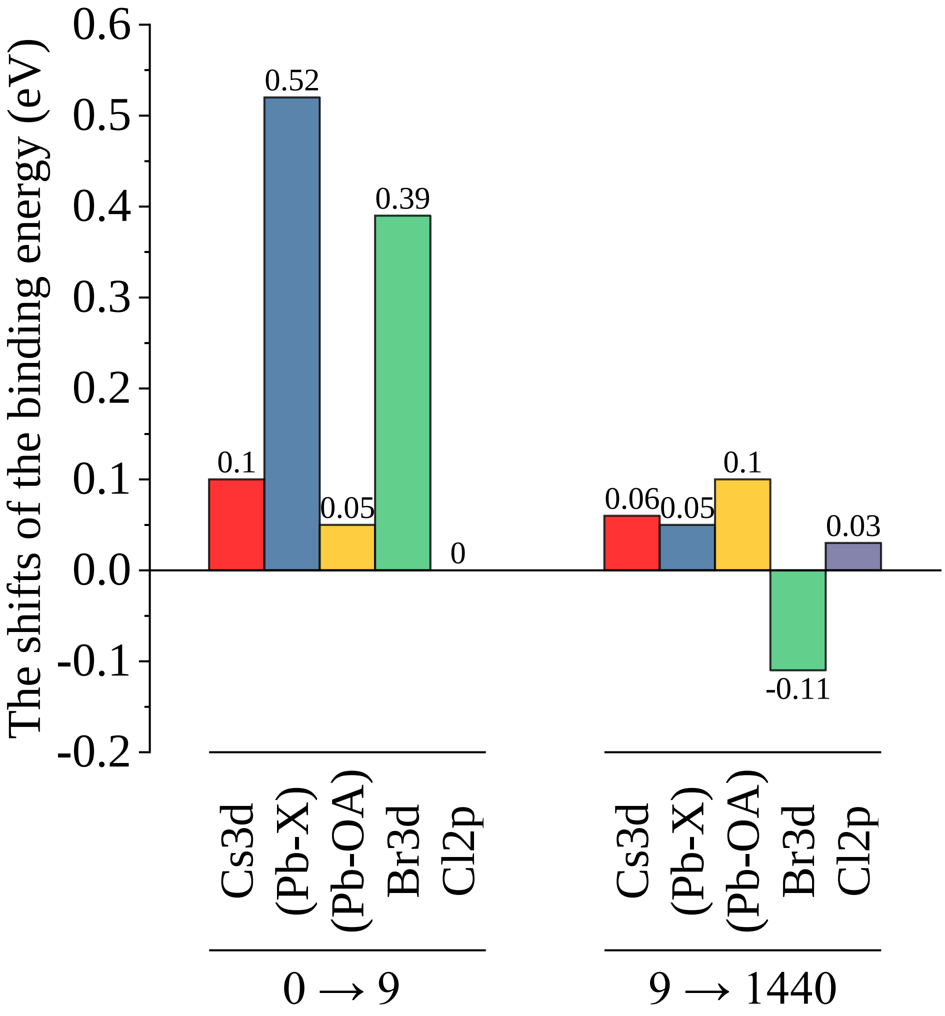

Figure S5. The shifts of the binding energy for various core levels by the time.

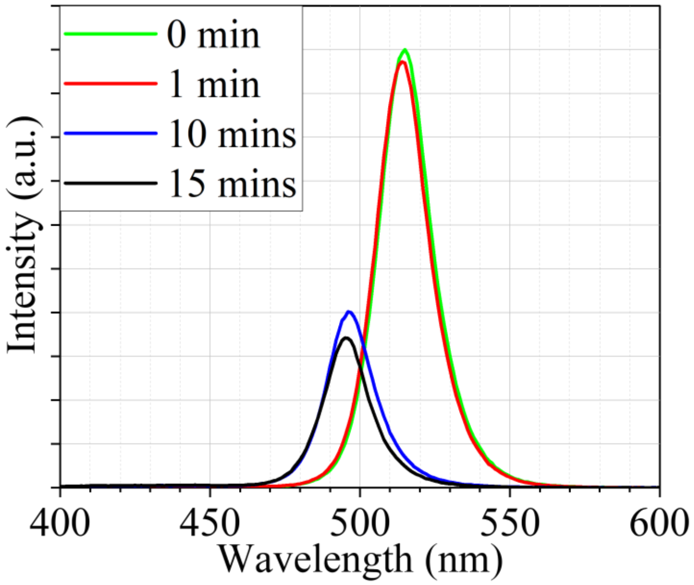

Figure S6. The PL results for the $CsPbBr_3$ PQDs thin film, which was exposed to 0.02 ppm of HCl gas for 1, 10, and 15 minutes to identify the optimal detection time.

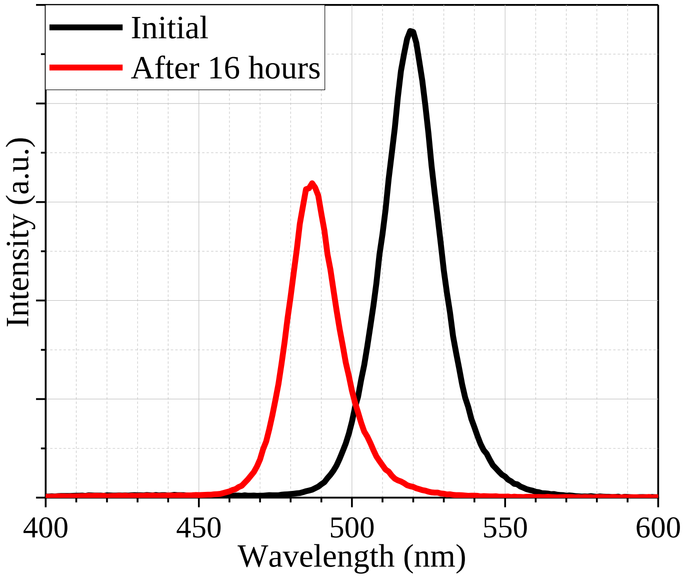

Figure S7. The PL results for the $CsPbBr_3$ PQDs thin film, which was exposed to 5% of NaOCl without UV-acceleration for 16 hours.

Table S1. Binding energy, full width at half maximum (FWHM), and spin-orbital splitting of Cs3d, Pb4f, Br3d, and Cl2p of $CsPbBr_3$ after exposed with HCl gas for 0, 9, and 1440 minutes.

**Samples**	**Core levels**		**Binding Energy (eV)**	**FWHM (eV)**	**Spin-Orbital Splitting (eV)**
**0 min**	Cs	$3d_{5/2}$	724.25	1.16	13.94
		$3d_{3/2}$	738.19	1.17	
	Pb-X	$4f_{7/2}$	138.14	1.26	4.86
		$4f_{5/2}$	143.00	1.39	
	Pb-OA	$4f_{7/2}$	138.05	0.85	4.86
		$4f_{5/2}$	142.91	0.82	
	Br	$3d_{5/2}$	68.00	1.01	1.05
		$3d_{3/2}$	69.05	1.07	
	Cl	2p	N/A	N/A	N/A
**9 mins**	Cs	$3d_{5/2}$	724.35	1.67	13.94
		$3d_{3/2}$	738.29	1.72	
	Pb-X	$4f_{7/2}$	138.66	1.75	4.86
		$4f_{5/2}$	143.52	1.78	
	Pb-OA	$4f_{7/2}$	138.10	1.01	4.86
		$4f_{5/2}$	142.96	0.94	
	Br	$3d_{5/2}$	68.39	1.62	1.05
		$3d_{3/2}$	69.44	1.81	
	Cl	$2p_{3/2}$	197.86	1.57	1.60
		$2p_{1/2}$	199.46	1.58	
**1440 mins**	Cs	$3d_{5/2}$	724.41	1.76	13.94
		$3d_{3/2}$	738.35	1.87	
	Pb-X	$4f_{7/2}$	138.71	1.92	4.86
		$4f_{5/2}$	143.57	1.89	
	Pb-OA	$4f_{7/2}$	138.20	1.03	4.86
		$4f_{5/2}$	143.06	1.00	
	Pb metal	$4f_{7/2}$	136.30	1.28	4.86
		$4f_{5/2}$	141.16	1.29	
	Br	$3d_{5/2}$	68.28	1.72	1.05
		$3d_{3/2}$	69.33	1.74	
	Cl	$2p_{3/2}$	197.89	1.49	1.60
		$2p_{1/2}$	199.49	1.48	

Table S2. Recently reported nanomaterial-based methods for detection of HCl gas.

Materials	Signal Type	LOD (ppm)	Response time (s)	Year	Ref.
Porphyrinated polyimide nanofibrous membrane	Fluorescence	5	10	2010	[1]
5,10,15,20-tetraphenylporphyrin contained in a poly(lactic acid) nanoporous fiber membrane	Absorbance/ Colorimetric	0.034	5	2016	[2]
Polyaniline Graft onto nylon fibers	Colorimetric	0.04	300	2018	[3]
ZnMOF	Fluorescence	10	5	2021	[4]
$CsPbBr_3$ PQDs	Fluorescence	5	300	2017	[5]
$CsPbBr_3$ nanowire	Fluorescence	5		2023	[6]
$CsPbBr_3$ PQDs	Fluorescence	0.005	600		This work

REFERENCES

1. Lv, Y.-Y.; Wu, J.; Xu, Z.-K., Colorimetric and fluorescent sensor constructing from the nanofibrous membrane of porphyrinated polyimide for the detection of hydrogen chloride gas. *Sens. Actuators, B* **2010,** *148* (1), 233-239.
2. Hu, M.; Kang, W.; Cheng, B.; Li, Z.; Zhao, Y.; Li, L., Sensitive and fast optical HCl gas sensor using a nanoporous fiber membrane consisting of poly(lactic acid) doped with tetraphenylporphyrin. *Microchim. Acta* **2016,** *183* (5), 1713-1720.
3. Thornton, B. T. E.; Harrison, A.; Pham, A. L.; Castano, C. E.; Tang, C., Polyaniline-Functionalized Nanofibers for Colorimetric Detection of HCl Vapor. *ACS Omega* **2018,** *3* (3), 3587-3591.
4. Zhu, Z.-H.; Ni, Z.; Zou, H.-H.; Feng, G.; Tang, B. Z., Smart Metal–Organic Frameworks with Reversible Luminescence/Magnetic Switch Behavior for HCl Vapor Detection. *Adv. Funct. Mater.* **2021,** *31* (52), 2106925.
5. Chen, X.; Hu, H.; Xia, Z.; Gao, W.; Gou, W.; Qu, Y.; Ma, Y., CsPbBr3 perovskite nanocrystals as highly selective and sensitive spectrochemical probes for gaseous HCl detection. *J. Mater. Chem. C* **2017,** *5* (2), 309-313.
6. Markina, D. I.; Anoshkin, S. S.; Masharin, M. A.; Khubezhov, S. A.; Tzibizov, I.; Dolgintsev, D.; Terterov, I. N.; Makarov, S. V.; Pushkarev, A. P., Perovskite Nanowire Laser for Hydrogen Chloride Gas Sensing. *ACS Nano* **2023,** *17* (2), 1570-1582.
